# 1+1 = 3: A Fusion of 2 Enzymes in the Methionine Salvage Pathway of *Tetrahymena thermophila* Creates a Trifunctional Enzyme That Catalyzes 3 Steps in the Pathway

**DOI:** 10.1371/journal.pgen.1000701

**Published:** 2009-10-23

**Authors:** Hannah M. W. Salim, Maria Cristina Negritto, Andre R. O. Cavalcanti

**Affiliations:** Biology Department, Pomona College, Claremont, California, United States of America; Washington University School of Medicine, United States of America

## Abstract

The methionine salvage pathway is responsible for regenerating methionine from its derivative, methylthioadenosine. The complete set of enzymes of the methionine pathway has been previously described in bacteria. Despite its importance, the pathway has only been fully described in one eukaryotic organism, yeast. Here we use a computational approach to identify the enzymes of the methionine salvage pathway in another eukaryote, *Tetrahymena thermophila*. In this organism, the pathway has two fused genes, *MTNAK* and *MTNBD*. Each of these fusions involves two different genes whose products catalyze two different single steps of the pathway in other organisms. One of the fusion proteins, mtnBD, is formed by enzymes that catalyze non-consecutive steps in the pathway, mtnB and mtnD. Interestingly the gene that codes for the intervening enzyme in the pathway, mtnC, is missing from the genome of *Tetrahymena*. We used complementation tests in yeast to show that the fusion of mtnB and mtnD from *Tetrahymena* is able to do in one step what yeast does in three, since it can rescue yeast knockouts of mtnB, mtnC, or mtnD. Fusion genes have proved to be very useful in aiding phylogenetic reconstructions and in the functional characterization of genes. Our results highlight another characteristic of fusion proteins, namely that these proteins can serve as biochemical shortcuts, allowing organisms to completely bypass steps in biochemical pathways.

## Author Summary

Fusion genes, composed of the complete sequence of two or more other genes, are excellent markers of evolution. In addition, fused genes are usually composed of genes with related functions, which makes them useful in inferring function when the function of one of their components is known. We detected a fusion gene in the eukaryotic organism *Tetrahymena thermophila* that, although composed of only two genes, seems to perform the function of three genes in this organism. To show that this is the case, we expressed the *Tetrahymena* fused gene in three different yeast strains, each lacking one of these three genes. The *Tetrahymena* gene was able to rescue the phenotype of all yeast strains, proving that it can perform the functions of the three genes in yeast. Our results highlight another important biochemical characteristic of fusion genes: they can serve as biological shortcuts, allowing a single fusion of two enzymes to functionally replace three independent enzymes.

## Introduction

The amino acid methionine and its derivative S-adenosylmethionine (SAM) are essential substrates in a variety of cellular reactions, including protein and nucleic acid methylation and polypeptide, polyamine and ethylene syntheses [1]–[5]. Ethylene, the hormone that regulates plant growth and development, is synthesized when the aminobutyrate group of SAM is released as 1-aminocyclopropane-1-carboxylic acid and then oxidized to form ethylene. SAM contributes to the synthesis of polyamines by providing the source of the groups spermidine and spermine [6]. The synthesis of polyamines and ethylene from the precursor SAM also results in the production of the byproduct 5′-methylthioadenosine (MTA), which can be converted back to methionine via the methionine salvage pathway.

The methionine salvage pathway is highly conserved in all domains of life [4], and its proper function is particularly important in cells that generate large quantities of polyamines and ethylene [7],[8]. While the complete methionine salvage pathway has been known for some years in bacteria [4],[5], only recently were all of the enzymes of the pathway characterized in an eukaryotic organism, the yeast *Saccharomyces cerevisiae* [9].

**Figure 1:**
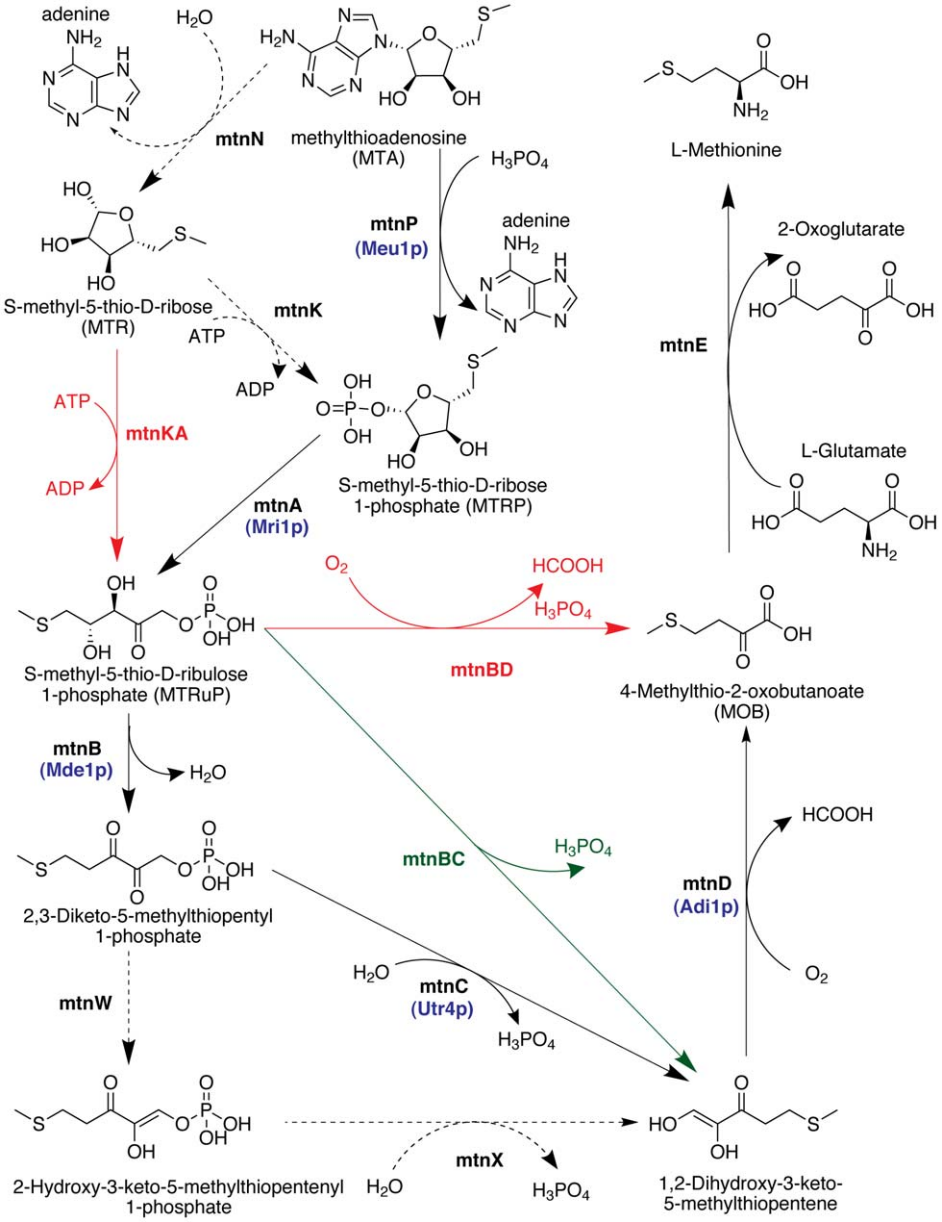
The methionine salvage pathway. The enzyme names are from [4], and compound names are from KEGG [18]. The reactions in black are known in bacteria [4]. The yeast pathway is indicated by blue gene names under the corresponding enzymes [9]. Dashed lines indicate variants of the pathway (see text). In [5] it was noted that the genes coding for mtnB and mtnC appear to be fused in *Arabidopsis thaliana*, and the genes for mtnB and mtnD appear to be fused in *Tetrahymena thermophila*, which indicates that the pathway in these organisms proceeds through the green and red reaction lines, respectively. We identified another fusion gene, between mtnK and mtnA, in *Tetrahymena* (red line).

Sekowska et al. [4] surveyed the complete genome sequences of several bacteria to determine the enzymes of the methionine salvage pathway (Figure 1). In bacteria there are two main variations in the pathway. Some bacteria, like *Pseudomonas aeruginosa*, convert MTA into S-methyl-5thio-D-ribose-1-phosphate (MTRP) in a single step using the enzyme MTA phosphorylase (MtnP; in this paper we will use the nomenclature for the enzymes in the pathway proposed in [4]). This same step is catalyzed by two separate enzymes in other bacteria, like *Bacillus subtillis*. In these organisms, mtnN, a hydrolase, first removes the adenine from MTA, converting it into S-methyl-5-thio-D-ribose (MTR). This compound is then converted into MTRP by the addition of a phosphate group by mtnK, a kinase.

The next step in the pathway involves the use of an isomerase, mtnA, to convert MTRP into S-methyl-5-thio-D-ribulose-1-phosphate (MTRuP). MTRuP is then dehydrated by a dehydratase, mtnB, generating 2,3-diketo-5-methylthiopentyl-1-phosphate.

In some bacteria, like *Klebsiella pneumoniae*, this diketone is converted into 1,2-dihydroxy-3-keto-5-methylthiopentene by an enolase-phosphatase, mtnC. In other organisms, like *Bacillus subtillis*, this reaction is carried out in two steps. The diketone is first converted into 2-hydroxy-3-keto-5-methylthiopentenyl-1-phosphate by an enolase, mtnW, and a phosphatase, mtnX, finally adds the phosphate group to the resulting molecule, forming 1,2-dihydroxy-3-keto-5-methylthiopentene.

The last two steps of the pathway are the formation of 4-methylthio-2-oxobutanoate (MOB) by the action of mtnD, a dioxygenase, followed by the transamination of this product to methionine. The last transamination step can be performed by many different transaminases depending on the organism [4],[9]. When a specific enzyme exists to catalyze this step, it is called mtnE; however, because of the variability of the enzymes that can potentially catalyze this step *in vivo*, we will use the term mtnE to refer to any transaminase that catalyses this last step. The whole pathway, including the two main variations, is depicted in Figure 1.

Pirkov et al. [9] used a computational and experimental approach to show that, in yeast, MTA is converted to MOB by the enzymes Meu1 (mtnP), Mri1 (mtnA), Mde1 (mtnB), Utr4 (mtnC) and Adi1 (mtnD). The final transaminase step can be catalyzed by several transaminases in yeast. For example, both the aromatic amino acid transaminases (Aro8 and Aro9) and the branched chain amino acid transaminases (Bat1 and Bat2) are capable of transaminating MOB into methionine [9]. For simplicity, in the remainder of this paper we will refer to the yeast enzymes using the pathway nomenclature proposed in [4]; thus we will refer to Meu1 as mtnP, Mri1 as mtnA, and so forth.

Sekowska et al. [4] and Ashida et al. [5] noted that in *Arabidopsis thaliana* mtnB and mtnC are coded for by a single gene and are most likely part of a fusion protein that performs both functions. We propose to call this protein mtnBC. Ashida et al. [5] also noted that the genes coding for mtnB and mtnD are fused in *Tetrahymena thermophila*, suggesting that a single protein can perform the functions of mtnB and mtnD in this organism. We propose to call this protein mtnBD.

It is important to stress that the alternative pathways described in [4] are catalyzed by different enzymes. Although mtnN and mtnK together catalyze the same reaction as mtnP, they share no sequence similarity with mtnP. The same is true for mtnW and mtnX. While together they catalyze the same reaction as mtnC, they share no sequence similarities with mtnC. The fused proteins in *Tetrahymena* and plants are different in that they contain the same domains as their corresponding components. For example, the N-terminus of the mtnBD enzyme in *Tetrahymena* shares a strong sequence similarity with the entire sequence of mtnB, while the C-terminus shares a strong similarity to all of the sequence of mtnD. The same is true for the mtnBC enzyme in *Arabidopsis*.

Here we investigated the methionine salvage pathway in *Tetrahymena thermophila*. We used similarity searches to show that in addition to the fusion of mtnB and mtnD, this organism has another fusion protein in the methionine salvage pathway, a fusion of the mtnK and mtnA enzymes (which we will refer to as mtnAK). Homologs to proteins catalyzing all the steps of the methionine salvage pathway are present in *Tetrahymena*, with the exception of mtnC, the protein that catalyzes the step between those of mtnB and mtnD in the pathway. The lack of an mtnC homolog in the *Tetrahymena* genome led us to hypothesize that the fusion protein mtnBD might perform the function of mtnC in addition to those of mtnB and mtnD in this organism. We performed experiments that show that this hypothesis is correct and that the fusion mtnBD is a trifunctional enzyme able to catalyze three steps in the salvage pathway, those of mtnB, mtnC and mtnD. The two identified fusions make *Tetrahymena* able to recycle MTA into methionine with the use of only four enzymes, as opposed to the six enzymes required by yeast or the eight required by *B. subtillis* (Figure 1).

## Results

### The methionine salvage pathway in *Tetrahymena*

We used the sequences of the methionine salvage pathway enzymes from yeast and *Bacillus subtillis* as queries in blastp searches to find homologs in *Tetrahymena*. Because the pathway is slightly different in these two organisms – yeast uses mtnP and mtnC, while *B. subtillis* uses mtnN and mtnK, and mtnW and mtnX (Figure 1) – some genes are unique to each organism. Table 1 shows the best blastp hit for each of the genes in *Tetrahymena*. Because the last step in the pathway can be catalyzed by many different transaminases in different organisms [4],[9], we did not search specifically for homologs of these genes in *Tetrahymena*. However, *Tetrahymena* has homologs to both Bat1 and Bat2 (TTHERM_00765280; e = 6e-79 to both Bat1 and Bat2) and to Aro8 and Aro9 (TTHERM_00140940; e = 2e-9 to Aro9 and 1e-5 to Aro8), shown to be the enzymes responsible for the last step of the pathway, the MOB transamination reaction, in yeast [9].

**Table 1:** Homologs of *B. subtillis* and yeast methionine salvage pathway enzymes in *Tetrahymena*.

Protein Name (GenBank accession)	Enzyme name (EC number)*	Source organism	GenBank accession number of best hit in *Tetrahymena*	evalue (score) of best hit in *Tetrahymena*
mtnN (NP_390605)	S-adenosylhomocysteine/5′-methylthioadenosine nucleosidase (EC:3.2.2.9)	*B. subtillis*	XP_001012703	2e-10 (63.5)
mtnK (NP_389239)	methylthioribose kinase (EC:2.7.1.100)	*B. subtillis*	XP_001031773	1e-66 (250)
mtnP (NP_013117)	5′-methylthioadenosine phosphorylase (EC:2.4.2.28)	Yeast	No significant hit	0.15 (34.3)
mtnA (NP_015443)	methylthioribose-1-phosphate isomerase (EC:5.3.1.23)	Yeast	XP_001031773	2e-53 (207)
mtnA (NP_389238)	methylthioribose-1-phosphate isomerase (EC:5.3.1.23)	*B. subtillis*	XP_001031773	1e-53 (207)
mtnB (NP_012558)	methylthioribulose-1-phosphate dehydratase (EC:4.2.1.109)	Yeast	XP_001025046	6e-41 (164)
mtnB (NP_389244)	methylthioribulose-1-phosphate dehydratase (EC:4.2.1.109)	*B. subtillis*	XP_001025046	4e-15 (78.6)
mtnW (NP_389242)	2,3-diketo-5-methylthiopentyl-1-phosphate enolase (EC:3.1.3.77)	*B. subtillis*	No significant hit	1.6 (31.6)
mtnX (NP_389243)	2-hydroxy-3-keto-5-methylthiopentenyl-1-phosphate phosphatase (EC:3.1.3.77)	*B. subtillis*	No significant hit	0.001 (40.8)
mtnC (NP_010876)	2,3-diketo-5-methylthiopentyl-1-phosphate enolase-phosphatase (EC:3.1.3.77)	Yeast	No significant hit	0.24 (32.7)
mtnD (NP_389245)	1,2-dihydroxy-3-keto-5-methylthiopentene dioxygenase (EC:1.13.11.53)	*B. subtillis*	XP_001025046	3e-15 (78.6)
mtnD (NP_013722)	1,2-dihydroxy-3-keto-5-methylthiopentene dioxygenase (EC:1.13.11.53)	Yeast	XP_001025046	3e-26 (114)

*Enzyme names and EC numbers are from KEGG (Kyoto Encyclopedia of Genes and Genomes [18].

The results from the blast searches suggest that *Tetrahymena* has homologs to mtnN, mtnK, mtnA, mtnB and mtnD (Table 1). A single *Tetrahymena* protein (XP_001031773) is the best hit to both mtnK and mtnA, and another protein (XP_001025046) is the best hit to both mtnB and mtnD. Further blast searches using each of these *Tetrahymena* proteins as queries showed that the C-terminus of XP_001031773 has strong sequence similarity to the sequence of mtnA and that its N-terminus has strong sequence similarity to the sequence of mtnK. The same is true for XP_001025046; its N-terminus has strong sequence similarity to the sequence of mtnB, and its C-terminus has strong similarity to the sequence of mtnD. These results indicate that mtnA and mtnK might form a fusion protein (mtnAK), and mtnB and mtnD might form another fusion protein (mtnBD) in *Tetrahymena*. To our knowledge, the fusion mtnAK has not been previously described in any organism.

To further verify if the genes identified as part of the methionine salvage pathway in *Tetrahymena* are true orthologs to the yeast and *B. subtillis* genes, we ran blastp searches against yeast and *B. subtillis* proteins in the RefSeq database using the *Tetrahymena* proteins as queries. In each case, the best hit to the *Tetrahymena* gene in the other genome was the original gene we used in the first search, indicating that all genes identified are reciprocal best blast hits – a good indication that the genes are orthologs. In the case of the fusion genes, the two best hits in yeast and *B. subtillis* were the components of the fusion, as expected.

The absence of a homolog to mtnP implies that *Tetrahymena* hydrolyzes the adenine from MTA using mtnN, instead of using mtnP to convert MTA into MTRP like yeast does. It seems that the resulting molecule could be converted directly to MTRuP since we identified what seems to be a fusion of the mtnK and mtnA enzymes. As previously described [5], mtnB and mtnD also seem to be fused in *Tetrahymena*. Surprisingly, we could not find *Tetrahymena* homologs to either mtnC, or to mtnW or mtnX, the enzymes responsible for converting 2,3-diketo-5-methylthiopentyl-1-phosphate into 1,2-dihydroxy-3-keto-5-methylthiopentene. The lack of homologs to this step in the pathway led us to hypothesize that the fusion of mtnB with mtnD might be able to convert MTRuP into MOB without the help of mtnC or mtnW-mtnX.

It should be noted that although the yeast mtnP protein doesn't bring any *Tetrahymena* hits, the human mtnP (accession: NP_002442) brings two *Tetrahymena* hits with evalues of 1×10^−14^ (XP_001020972) and 2×10^−10^ (XP_001020274), respectively. These *Tetrahymena* genes, however, are other types of purine nucleoside phosphorylases. XP_001020972 is an inosine and guanosine-specific phosphorylase; its best hit in humans is to a nucleoside phosphorylase (NP_000261; evalue = 2×10^−61^) and not to mtnP (evalue = 1×10^−14^); its best hit in yeast is to NP_013310 (evalue = 4×10^−48^) and not to mtnP (evalue = 0.019). The best hits of the other *Tetrahymena* nucleoside phosphorylase, XP_001020274, are NP_013310 in yeast (evalue = 5×10^−45^) and NP_000261 (evalue = 5×10^−54^) in humans, and not the mtnP enzymes in these organisms (yeast mtnP, evalue = 2.5; human mtnP, evalue = 2×10^−10^). Thus, it seems that although *Tetrahymena* has at least two purine nucleoside phosphorylase proteins, neither of these share functional homology with mtnP (they are not best reciprocal blast hits).

### The mtnAK fusion protein

The results of our blast searches suggested that the mtnK and mtnA enzymes are fused in *Tetrahymena*, as both proteins hit the same *Tetrahymena* gene. Based on the nomenclature proposed by Sekowska et al. (2004), we will refer to this enzyme as mtnAK. The *Tetrahymena* protein is 779 amino acids long. Approximately the first 350 amino acids hit mtnA; mtnA in *B. subtillis* is 353 amino acids long, and its alignment to the N-terminus of mtnAK has a percent identity of 38% and spans residues 17 to 353. The second half of mtnAK hits mtnK; mtnK in *B. subtillis* is 397 amino acids long, and its alignment to the C-terminus of mtnAK has a percent identity of 38% and spans residues 30 to 393.

**Figure 2:**
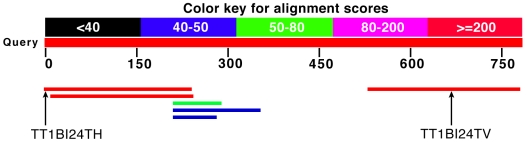
Screenshot of a tblastn search of the mtnAK enzyme from *Tetrahymena* (XP_001031773) against the EST sequences from *Tetrahymena* in GenBank. The EST sequences TT1BI24TH (acc: FF565362; evalue = 1×10^−125^) and TT1BI24TV (acc: FF565363; evalue = 2×10^−125^) correspond to the 5′- and 3′-end of a single cDNA clone, indicating that the fusion protein is expressed in *Tetrahymena*.

To determine whether the *Tetrahymena* protein XP_001031773 is really a fusion of mtnK and mtnA or just an artifact of incorrect gene prediction, we performed a tblastn search of the protein sequence of mtnAK against the NCBI EST database for evidence that this protein is expressed in *Tetrahymena*. The *Tetrahymena* EST sequences TT1BI24TH (acc: FF565362) and TT1BI24TV (acc: FF565363) correspond to the 5′ and 3′ of a single cDNA clone and hit the N- and C-termini of the mtnAK fusion protein, with evalues of 1×10^−125^ and 2×10^−125^, respectively (Figure 2). This strongly suggests that the genes coding for mtnK and mtnA are fused in *Tetrahymena* and code for an expressed fused protein, with the N-terminus corresponding to mtnA and the C-terminus corresponding to mtnK.

We used the sequence of the mtnAK protein in *Tetrahymena* to search for other organisms that might have this fusion in the NR database from GenBank using blastp. The same fusion of mtnK and mtnA is present in at least some stramenopile and plant species like *Ostreococcus tauri*, *Phaeodactylum tricornutum*, *Micromonas pusilla* and *Micromonas sp. RCC299*. Interestingly, in both sequenced *Micromonas* genomes this gene is annotated as a fusion of a translation initiation factor with methylthioribose kinase (mtnK). This is due to the fact that methylthioribose-1-phosphate isomerase (mtnA) shares sequence similarities with eIF-2B-alpha [4]. However, because it is fused with another gene in the methionine salvage pathway and has strong sequence similarity with mtnA, this gene is most likely a fusion between mtnK and mtnA and not a translation initiation factor.

### The mtnBD fusion protein

Our blast searches confirmed the previously described fusion of mtnB and mtnD in *Tetrahymena* [5]; we will refer to this enzyme as mtnBD. To determine if this is a real fusion enzyme or just a gene prediction artifact, we searched the *Tetrahymena* ESTs in GenBank for evidence of transcripts of this gene.

The searches did not return any *Tetrahymena* EST with significant similarity to the mtnBD fusion protein. Therefore, to confirm that mtnBD is a real fusion protein in *Tetrahymena*, we used a *Tetrahymena* cDNA library to determine the presence of a transcript of the predicted gene coding for the fusion protein. PCR results (not shown) indicated that the gene is transcribed as a single mRNA in *Tetrahymena*, and thus codes for an expressed fusion protein.

We used the sequence of the mtnBD gene from *Tetrahymena* to search the NR database from GenBank in order to identify other organisms that might have this same gene fusion. Interestingly, this fusion gene is not present in any other organism in GenBank and is absent even in ciliate species closely related to *Tetrahymena*, like *Paramecium tetraurelia*. This species has a fully sequenced genome that completely lacks homologs to any enzyme in the methionine salvage pathway.

### Functional analysis of the mtnBD fusion

Sequence similarities to mtnB and mtnD and the lack of an mtnC homolog suggest that the fusion protein, mtnBD, might be able to perform the functions of mtnC in addition to those of mtnB and mtnD in *Tetrahymena*. To determine if this hypothesis was correct, we investigated the *in vivo* function of the *T. thermophila* fused gene by doing complementation studies in yeast cells.

We designed a synthetic gene that codes for the same amino acid sequence as the mtnBD fusion from *Tetrahymena*, but with codon usage optimized for expression in yeast cells. This gene will be referred to as *SYN-MTNBD*. We cloned *SYN-MTNBD* under the control of a *GAL* promoter in the pGREG505 plasmid (Euroscarf).

The plasmid (pGREG505/SYN-MTNBD) was transformed into three different *S. cerevisiae met^−^* strains (*met15Δ0*) that had the genes that code for the enzymes mtnB, mtnC and mtnD of the methionine salvage pathway individually deleted. The goal was to determine if the *SYN-MTNBD* gene is capable of complementing each single knockout strain. As a negative control each strain was also transformed with the parent vector, pGREG505.

Although *met^−^* yeast strains usually cannot grow in media lacking methionine, they can grow if the media is supplemented with MTA at a concentration of at least 5 mM [9],[10]. In such media, yeast cells are able to synthesize methionine from MTA using the methionine salvage pathway. The three deletion strains used in this experiment cannot grow in the absence of methionine even when the medium is supplemented with MTA since each strain lacks a gene essential to the functioning of the methionine salvage pathway and is unable to synthesize methionine from MTA (Figure 1).

If our hypothesis was correct, and the mtnBD fusion replaces mtnB, mtnC and mtnD in *Tetrahymena*, a synthetic version of the *T. thermophila* mtnBD fusion protein would restore the methionine salvage pathway in the three yeast deletion strains. We predicted that, while the three deletion strains would fail to grow in media lacking methionine but containing MTA due to the deletions in the methionine salvage pathway, the three strains would grow in this media when the *SYN-MTNBD* gene is expressed.

**Figure 3:**
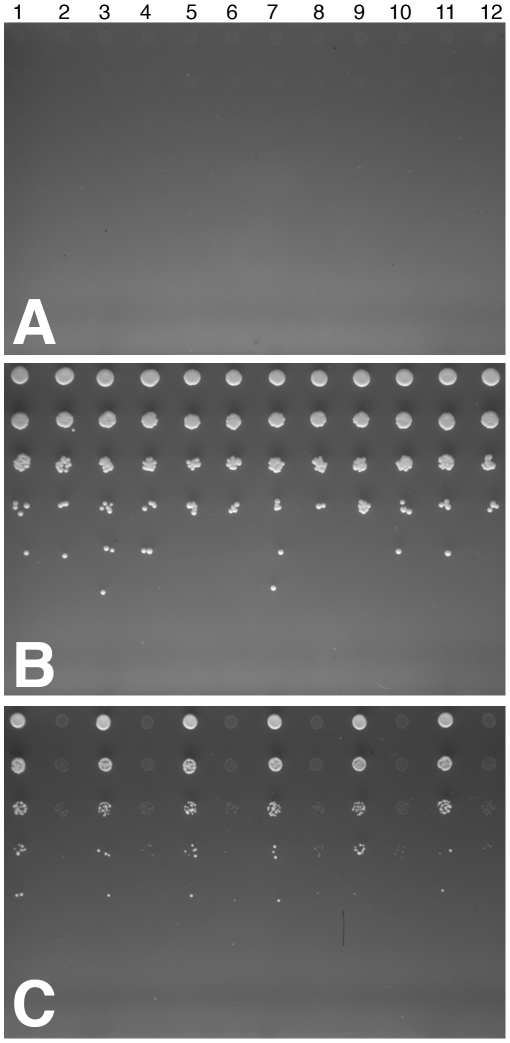
Complementation experiment of yeast single knockout strains with mtnBD fusion gene from *Tetrahymena*. Cells were grown to late exponential phase in −Leu +Met liquid media, transferred to −Leu−Met for an overnight to deplete internal Methionine pool, and serial dilutions for each strain were prepared in a 96-well plate with 1×10^8^ cells/ml, 1×10^7^ cells/ml, 1×10^6^ cells/ml, 1×10^5^, 1×10^4^ cells/ml, and 1×10^3^ cells/ml. 3 µl of each diluted culture was spotted with a 96-well pin replicator onto (A) −Met−MTA (negative control plate). (B) +Met plates, (positive control). (C) −Met +MTA (5 mM) (experimental plate). The strains assayed are: 1. *mtnB*Δ + pGREG505/*SYN-MTNBD*; 2. *mtnB*Δ + pGREG505; 3. *mtnC*Δ + pGREG505/*SYN-MTNBD*; 4. *mtnC*Δ + pGREG505; 5. *mtnD*Δ + pGREG505/*SYN-MTNBD*; 6. *mtnD*Δ + pGREG505; columns 7–12 are replicates of columns 1 through 6. After four days, none of the six yeast strains grew on the negative control plate. All six strains grew on the positive control plate. Only the strains transformed with pGREG/*SYN-MTNBD* (columns 1, 3, 5, 7, 9, 11) grew in the experimental plate.

Figure 3 shows the results of the experiment. None of the strains grew on the negative control plate (Figure 3A: −Met−MTA medium; 4 days); and all strains grew on the positive control plate (Figure 3B: +Met medium; 4days). Only the deletion strains transformed with the pGREG505/*SYN-MTNBD* plasmid grew on the experimental plate; the deletion strains transformed with the control parent vector (pGREG505) did not grow (Figure 3C: −Met+MTA; 4 days).

As expected, the growth of the strains transformed with *SYN-MTNBD* was slower in the medium supplemented with MTA than in the +Met medium. Yeast strains with a similar genetic background grow slower in medium supplemented with MTA than in medium supplemented with methionine, even when they have an intact methionine salvage pathway [9]. This is probably a result of having to synthesize the methionine necessary for growth from MTA, instead of having it readily available in the medium.

Our results show that a single fusion protein in the methionine salvage pathway of *T. thermophila*, mtnBD, accomplishes enzymatic functions that are generally performed by three enzymes, mtnB, mtnC and mtnD, in other organisms.

## Discussion

In *Tetrahymena* the conversion of MTA to methionine seems to be catalyzed by four enzymes. The first, mtnN, is a hydrolase that converts MTA into MTR. The second is a fusion of the mtnK and mtnA proteins, mtnAK, that converts MTR into MTRuP. The third is a fusion of the mtnB and mtnD proteins, mtnBD, that converts MTRuP into MOB. Finally, although we cannot determine the identity of the fourth protein in the pathway, the MOB transaminase (mtnE), *Tetrahymena* has homologs to both Bat1/Bat2 and Aro8/Aro9, the proteins known to perform this step in yeast [9].

It is interesting that *Tetrahymena* does not have a homolog either to mtnC or to mtnX and mtnW, the enzymes that convert 2,3-diketo-5-methylthiopentyl-1-phosphate into 1,2-dihydroxy-3-keto-5-methylthiopentene. Because of the lack of these enolase and phosphatase enzymes, we hypothesized that the mtnBD fused enzyme would be able to catalyze the conversion of MTRuP into MOB, completely bypassing the enolase-phosphatase reaction needed in other organisms.

We used yeast strains with deletions of the *mtnB*, *mtnC*, or *mtnD* genes to test if the mtnBD fusion protein from *Tetrahymena* is able to catalyze the three steps of the pathway. Our results show that the *Tetrahymena* fusion enzyme complements all three yeast deletion strains and thus is capable of catalyzing the three steps of the pathway.

Fusion genes usually involve genes of related function [11],[12]. One reason for this could be that a fusion simplifies the regulation of a pathway by automatically co-expressing and co-localizing the fused genes. In *Tetrahymena*, the presence of two fused genes simplifies the regulation of the methionine salvage pathway; the number of independently regulated genes in this pathway in *Tetrahymena* is only four, as opposed to eight in *B. subtillis* and six in yeast. Because the transaminase reaction is usually performed by recruiting an enzyme with more than one substrate specificity, this enzyme shouldn't be expected to be co-regulated with the remainder of the pathway. As such, *Tetrahymena* only needs to coordinate the expression of three genes to recycle methionine from MTA.

Fusion genes are also important phylogenetic markers. As an example, the fusion proteins dihydrofolate reductase/thymidylate synthase and carbamoyl phosphate synthase/dihydroorotase/ aspartate carbamoyltransferase were used to pinpoint the root of the eukaryotic tree [13]–[15]. We performed blast searches to determine the phylogenetic distribution of the fusion mtnBD. Unfortunately, these searches revealed that *Tetrahymena* is the only organism in which the mtnBD fusion is present, as no other sequence in the NR database has hits to the two proteins.

In addition, it is interesting to note that, although the methionine pathway is present and highly conserved in all domains of life, it is absent from most species closely related to *Tetrahymena*. Only two ciliate genomes have been completely sequenced thus far. The pathway is present in *Tetrahymena*, but completely absent in the other sequenced ciliate genome, that of *Paramecium tetraurelia*. Ciliates, together with apicomplexans and dinoflagellates, compose the alveolate clade. There are no sequenced dinoflagellate genomes, but according to blast searches the pathway is also absent from all apicomplexan genomes available (in August 2009 the genomic blast from NCBI allowed blastp searches of the genomes of 14 apicomplexans: 6 *Plasmodium* species, 3 *Cryptosporidium* species, 2 *Theileria* species, *Babesia bovis*, *Eimeria tenella* and *Toxoplasma gondii*; all these genomes lack the pathway). This makes *Tetrahymena* unique among alveolates and could lead to a better understanding of the pathway and of why other alveolates eliminated the pathway.

The absence of the mtnBD fusion protein in other organisms limits its use as a phylogenetic marker. The mtnAK fusion, on the other hand, is more interesting from a phylogenetic standpoint. Even though a full analysis of the phylogenetic distribution of this fusion protein is beyond the scope of this paper, we noticed that, in addition to *Tetrahymena*, the fusion is present in several stramenopile and plant genomes, but not in any other genome. Ciliates, like *Tetrahymena*, alveolates and stramenopiles, form the chromalveolate group, thought to be a sister group of plants.

### Fusion genes as biochemical shortcuts

Interestingly, the fusion of mtnB and mtnD in *Tetrahymena* led to a gain of function of the fused protein, as evidenced by its ability to complement yeast *mtnB*, *mtnC*, or *mtnD* single knock-out strains. This demonstrates another potential advantage of fused proteins, the ability to short-circuit biochemical pathways. It is worth noting that mtnC can catalyze in one step a reaction that in other organisms is performed in two steps by mtnX and mtnW. In these organisms we could say that 1+1 = 4, and that the fusion of two genes in *Tetrahymena* generates a tetrafunctional protein. Because mtnBD is able to catalyze the reaction of mtnC, this protein probably became superfluous in *Tetrahymena* and was eliminated during evolution.

It is possible that in order for mtnBD to catalyze the enolase-phosphatase reaction of mtnC, another enolase-phosphatase, non-homologous to mtnC or to mtnW/mtnX, is needed. We believe this scenario to be highly unlikely, however, since this other enolase-phosphatase would have to be present in yeast and only functional when mtnBD is being expressed, as the absense of mtnC is lethal for yeast of this genetic background in −Met+MTA medium in the absense of mtnBD. Furthermore, in all eukaryotes in which we searched for the enzymes of the pathway using blast, either mtnC or mtnW/mtnX is present, with the exceptions of *Tetrahymena* and organisms that completely lack the pathway. Because *Tetrahymena* is the only organism in which the fusion gene is present and in which mtnC and mtnW/mtnX are missing, and because the fusion is able to complement the deletion of the enolase-phosphatase (mtnC) in a heterologous system, yeast, a much more likely explanation is that the *Tetrahymena* fusion gene somehow bypasses the enolase-phosphatase step or is able to catalyze it.

It is important to stress that the fusion protein in *Tetrahymena* is composed of only the sequences of mtnB and mtnD, with a linker of about 10–15 amino acids in between. The fusion has no significant sequence similarity with either mtnC or mtnW/mtnX. It would be interesting to characterize how mtnBD allows the cell to bypass the enolase-phosphatase step in the pathway, or how it catalyzes this step, and to compare its mechanism to the mechanism of mtnC or mtnW/mtnX.

MtnD is an interesting dioxigenase. *In vivo*, this enzyme catalyzes different reactions depending on the metal ion bound to the active site [16],[17]. When bound to Ni^2+^ the enzyme catalyzes the formation of 3-Methylthiopropionate, formate and carbon monoxide, while when bound to Fe^2+^ it catalyzes the formation of 4-Methylthio-2-oxobutanoate and formate. Only when it is bound to Fe^2+^ is the enzyme involved in the methionine salvage pathway, as 4-Methylthio-2-oxobutanoate (MOB) is the substrate of mtnE in the last step of the pathway. The purpose of the off-pathway reaction is not known, and the product 3-Methylthiopropionate is cytotoxic [17]. It would be interesting to determine if the fusion enzyme mtnBD, besides being able to catalyze the three reactions in the methionine pathway, still retained the ability to bind Ni^2+^ and generate 3-Methylthiopropionate and carbon monoxide. It would also be interesting to determine if the mtnBD gene is more efficient than the combined three genes it replaces, mtnB, mtnC and mtnD, and whether it leads to more efficient methionine recycling in *Tetrahymena*.

## Materials and Methods

### Identification of homologs of the methionine pathway in *Tetrahymena*

The complete set of enzymes of the methionine salvage pathway has been described in various bacteria [3],[4] and recently in *S. cerevisiae* [9]. We used the KEGG database (Kyoto Encyclopedia of Genes and Genomes [18]) to download the sequences of the methionine salvage pathway enzymes in *Bacillus subtillis* and yeast.

In *B. subtillis* MTA is converted into methionine by mntN, mtnK, mtnA, mtnB, mtnW, mtnX, mtnD and a transaminase. In yeast the pathway involves mtnP, mtnA, mtnB, mtnC, mtnD and a transaminase. Because the last step of the pathway can be performed by a variety of transaminase enzymes, we excluded it from the analyses. Using each of the 7 *B. subtillis* enzymes and each of the 5 yeast enzymes as the queries, we performed blastp searches [19] of all the *Tetrahymena* protein sequences in RefSeq [20]. To eliminate the possibility that a homolog for a given protein exists in *Tetrahymena* but has not been annotated, we used tblastn to search the *Tetrahymena* genome sequence, using as queries each of the proteins that didn't return a significant hit in the blastp searches. None of these returned significant hits.

### In vivo characterization of the *mtnBD* gene

#### Design of the *syn-mtnBD* gene

Because *Tetrahymena* uses an alternative genetic code where TAA and TAG code for glutamine instead of a stop codon [21], we had to modify the nucleotide sequence for expression in yeast. We changed each TAA and TAG codon in the nucleotide sequence to a glutamine codon (CAA or CAG). In addition, we optimized the codon usage of the gene for expression in yeast, added a yeast initiation context (TTCAAACAAA) upstream of the start codon, and added appropriate restriction sites upstream and downstream of the gene. We purchased a synthetic version of this construct from GenScript Corporation (Piscataway, NJ). The synthetic version of the gene will be referred as *SYN-MTNBD* in the paper.

### Strains and plasmid construction

In yeast, the genes coding for mtnB, mtnC and mtnD were identified by Pirkov et al. (2008) as, *MRI1* (*YPR118W*), *MDE1* (*YJR024C*) and *UTR4* (*YEL038W*), respectively. The single null mutant strains used in this study, *mri1Δ*, *mde1Δ* and *utr4Δ* (referred to in this study as *mtnBΔ*, *mtnCΔ*, and *mtnDΔ*, respectively) and a WT strain, all in the BY4741 genetic background (MATa *his3Δ1 leu2Δ0 met15Δ0 ura3Δ0*), were purchased from the single gene knockout collection from Open Biosystems.

The synthetic gene (*SYN-MTNBD*) was synthesized and cloned into *E. coli* pUC57-simple expression vector by GeneScript Corporation. pUC57/*SYN-MTNBD* was digested with NotI and NheI, and the DNA fragment with the *SYN-MTNBD* gene was cloned into yeast expression vector pGREG505 (purchased from EUROSCARF Frankfurt, Germany), cut with the same set of enzymes to create pGREG505/*SYN-MTNBD*. pGREG505/*SYN-MTNBD* has a *LEU2* selectable marker and the *SYN-MTNBD* gene is under the control of the *GAL* promoter.

pGREG505/*SYN-MTNBD* and pGREG505 were transformed into each of the three yeast deletion strains *mtnBΔ*, *mtnCΔ* and *mtnDΔ*. Transformants were selected by plating on synthetic media lacking Leucine (−Leu plates). Leu+ transformants were picked and re-streaked to single colonies on −Leu plates. Liquid cultures were then inoculated and grown to saturation to generate frozen stock cultures of the transformed null mutant strains with pGREG505/*SYN-MTNBD* and pGREG505.

### Cultures conditions and media

All *Saccharomyces cerevisiae* strains were maintained and grown in YPD (yeast Nitrogen base, peptone, dextrose) minimal media or the appropriate drop-out medium as specified in Pirkov et al. 2008 (1.7 g/L yeast nitrogen base without amino acids and ammonium sulfate, 0.5 g/L serine, 0.2 g/L aspartate, 0.17 g/L tryptophan, 0.12 g/L adenine, 0.1 g/L leucine, 0.02 g/L tyrosine, and 0.05 g/L of histidine, uracil, lysine, alanine, phenylalanine, tyrosine, isoleucine, valine and tyrosine) with 2% glucose or galactose. Drop-out media were made by leaving out the corresponding amino acid or base. Since all strains used are *met15Δ0*, they cannot grow in the absence of methionine (Met). The media of the positive control plates used in the complementation studies were supplemented with 5 mM Met, and the experimental plates were supplemented with 5 mM MTA (Sigma-Aldrich, St. Louis, MO). When expression of the cloned gene was necessary, glucose was replaced with galactose. Strains carrying the pGREG505 plasmids (*LEU2* marker) were grown in −Leu drop-out media to prevent loss of the plasmids.

### Complementation tests

The three yeast deletion strains *mtnBΔ*, *mtnCΔ* and *mtnDΔ* transformed with pGREG505/*SYN-MTNBD* or pGREG505 were streaked to single colonies on −Leu +Met agar plates to select for retention of the plasmids. Liquid cultures in 1 ml −Leu +Met were started by inoculating with a single colony and grown overnight in a roller drum at 30°C. Cells from the overnight cultures where spun down, washed, resuspended in −Leu −Met medium and put back for another overnight in the roller drum at 30°C. This step was performed to exhaust extracellular and intracellular pools of methionine [9]. After an overnight in −Leu −Met medium the number of cells/ml in each liquid culture was determined by counting with a hemacytometer. 50 µl serial dilution samples of each strain with 1×10^8^ cells/ml, 1×10^7^ cells/ml, 1×10^6^ cells/ml, 1×10^5^ cells/ml, 1×10^4^ cells/ml, 1×10^3^ cells/ml, 1×10^2^ cells/ml and 1×10 cells/ml were transferred onto a 96-well plate. 3 µl samples were then pinned onto agar plates in triplicates using a 96-well floating Pin Replicator (V&P Scientific, Inc., San Diego, CA). Agar plates used: +Gal −Leu −Met in the negative controls, +Gal −Leu +Met in the positive controls, and +Gal −Leu −Met +MTA in the experimental plates. Pinned cells were grown at 30°C for four days and then visually analyzed and photographed with a Kodak IC440 imaging system (Figure 2).

Because the strains are met15Δ0, they cannot grow in the absence of methionine. Pirkov et al. [9] showed, however, that if supplemented with enough MTA these strains are able to grow in the absence of methionine by converting the MTA into methionine by means of the methionine salvage pathway. Each of our deletion strains has one of the genes in the pathway deleted and cannot convert MTA to methionine, and thus should not be able to grow in the presence of MTA, unless the *SYN-MTNBD* gene complements the gene deleted in the strain. All strains should grow in media supplemented with methionine (positive control), and none of the strains should grow in −Met −MTA media (negative control).
